# Two‐thirds return but performance suffers: ACL reconstruction outcomes across elite European women's soccer leagues

**DOI:** 10.1002/jeo2.70888

**Published:** 2026-08-17

**Authors:** Haneef Khan, Forest Rodriguez, Amina Khan, Ashley Rosenberg, Stephen Marcaccio

**Affiliations:** ^1^ University of Michigan Ann Arbor Michigan USA; ^2^ Department of Orthopaedics The Warren Alpert Medical School of Brown University Providence Rhode Island USA; ^3^ University Orthopedics Inc. East Providence Rhode Island USA; ^4^ Sidney Kimmel Medical College Philadelphia Pennsylvania USA

**Keywords:** athletic performance, career outcomes, knee injury, ligament, rehabilitation

## Abstract

**Purpose:**

To analyse return‐to‐play (RTP) rates, timing and performance outcomes following anterior cruciate ligament (ACL) reconstruction (ACLR) in elite European women's soccer players across the top five leagues.

**Methods:**

We reviewed ACL injuries sustained by female professional soccer players across the Bundesliga, Women's Super League, Primera División Femenina, Serie A Women and Première Ligue between 2007 and 2025. Players were included if they had a confirmed ACL injury requiring ACLR and one season of performance data pre‐ and post‐injury. Players were excluded if the injury occurred during their rookie season or after the 2023 season. Data were collected from Soccerdonna.de and Transfermarkt.

**Results:**

A total of 242 ACL injuries in 206 unique players were included, with a median follow‐up of 2.0 seasons (interquartile range [IQR]: 1.0–4.0). The cohort had a median age at injury of 22.7 years (IQR: 20.4–26.0; mean 23.4 ± 3.8 years). Of 242 injuries, 161 (66.8%) resulted in RTP. For those who returned, the median RTP time was 9.8 months (IQR: 7.9–11.4). Only 7 (5.1%) players achieved RTP before 6 months. In the first year following RTP, significant performance decreases were observed: games played (−47.4%, *p* < 0.001), total minutes (−57.8%, *p* < 0.001) and minutes per game (−24.4%, *p* < 0.001). Midfielders experienced the greatest decline in playing time (−41.2%, *p* < 0.001), while goalkeepers showed minimal change (−8.0%, *p* = 0.625). No significant age‐related differences were observed. Median post‐injury career length was 2.0 seasons (IQR: 2.0–4.0).

**Conclusions:**

A RTP rate of 66.8% was observed following ACLR in elite European women's soccer; however, returning players faced significant performance decreases in the first year post‐return, with notable position‐specific variations. These reductions likely reflect a combination of injury‐related recovery factors and team selection dynamics. Rehabilitation and RTP strategies should therefore integrate position‐specific demands, realistic performance expectations and gradual reintegration plans, particularly for midfielders who demonstrated the greatest performance decline.

**Level of Evidence:**

Level III.

**Abbreviations**
ACL: anterior cruciate ligament
ACLR: anterior cruciate ligament reconstruction

## INTRODUCTION

Anterior cruciate ligament (ACL) injuries represent one of the most significant concerns in women's professional soccer, with female athletes experiencing injury rates 2–8 times higher than their male counterparts [9]. The growing professionalization of women's soccer has intensified scrutiny of these injuries, given their impact on athletic careers and long‐term knee health [10]. Epidemiological studies report injury rates exceeding 30 per 1000 player‐hours in some elite leagues, underscoring the scale of the problem [18]. Heightened susceptibility in female athletes has been attributed to anatomical, biomechanical and hormonal factors, including smaller ACL dimensions, increased knee valgus moments and menstrual‐cycle‐related fluctuations in ligament laxity [14, 19, 30].

Following ACL reconstruction (ACLR), return‐to‐play (RTP) criteria have shifted from time‐based toward criteria‐based protocols incorporating strength testing, functional movement analysis and psychological readiness [3, 17, 24, 28, 33]. Optimal RTP timing remains debated; returns before 9 months are associated with elevated reinjury risk, and biological graft maturation continues well beyond 6 months postoperatively [11, 26]. Despite these advances, comprehensive multi‐league data on RTP rates and subsequent performance outcomes in elite women's soccer remain limited—most published studies focus on single leagues or small cohorts [1, 4].

The primary aim of this study was to determine RTP rates and timing following ACLR across the top five European women's professional leagues, hypothesizing that a majority of players would successfully achieve RTP with RTP timing consistent with current best‐practice rehabilitation recommendations. Secondary aims were to (1) analyse performance changes in the two years following RTP, hypothesizing significant declines relative to pre‐injury levels; (2) identify position‐ and age‐related factors influencing outcomes, hypothesizing greater deficits in field positions than goalkeepers and (3) assess career longevity following ACLR, hypothesizing a median career continuation of fewer than four seasons post‐RTP.

## METHODS

### Study design and setting

This study was conducted in accordance with applicable ethical standards. As only publicly available data from established sports statistics databases were used, no patient‐identifiable information was accessed, and no institutional ethics board approval was required under applicable regulations.

This retrospective cohort study analysed ACL tears in female soccer players from five major European leagues: Bundesliga (Germany), Women's Super League (England), Primera División Femenina (Spain), Serie A Women (Italy) and Première Ligue (France). Data were collected from Soccerdonna.de for injury records and Transfermarkt for season performance statistics, covering the period from 2007 to 2025.

### Participants

Inclusion criteria comprised: (1) female professional soccer players competing in one of the five target leagues; (2) confirmed ACL injury requiring ACL reconstruction (ACLR); (3) no history of revision ACLR, contralateral ACL injury or concomitant ligament, cartilage or meniscal injury requiring surgical management; (4) minimum one complete season of performance data available both pre‐ and post‐injury, with the post‐injury period beginning following ACLR. Players were excluded if (1) the ACL injury occurred during their rookie season, precluding establishment of a pre‐injury performance baseline or (2) the injury occurred after the 2023 season, providing insufficient follow‐up time to assess RTP.

The primary performance comparison required a minimum of two complete seasons pre‐ and post‐injury (*n* = 90). Players with fewer than two available seasons were included in secondary analyses only, where noted. The injury season itself was excluded from all pre–post performance comparisons to avoid confounding by partial participation.

### Data collection

ACL injuries were identified through multiple sources, including official league injury reports, club medical announcements, media reports and player social media disclosures. Each injury was verified through at least two independent sources, specifically official club medical announcements cross‐referenced against established sports injury tracking databases (Soccerdonna.de and Transfermarkt). Player demographic data, including age, nationality, position and league affiliation, were collected from official league databases and verified through club records.

Performance data were collected for a minimum of two seasons before injury and two seasons following RTP, when available. Missing seasons due to career length, transfers or incomplete records were not imputed. RTP was defined as participation in an official league match for a minimum of one minute. RTP timing was calculated as the interval between the date of ACLR and the first competitive match participation. Career longevity was assessed as the number of complete seasons played following RTP.

The primary outcome was minutes per game in the first complete season post‐RTP. Secondary outcomes were games played per season and total minutes per season.

### Statistical analysis

Statistical analyses were performed using Python version 3.14 (Python Software Foundation). Players who returned to play were stratified by age at injury using a 25‐year cutoff, selected a priori based on prior literature suggesting this threshold corresponds to a period of peak physical performance and increased biological recovery capacity in professional female athletes [21, 23]. Given the non‐normal distribution of most variables, data are presented as median with interquartile range (IQR) unless otherwise specified.

Normality of continuous variables was assessed using the Shapiro–Wilk test. Given the non‐normal distribution of performance metrics, non‐parametric tests were employed throughout. Paired pre–post comparisons used the Wilcoxon signed‐rank test. Independent group comparisons (age groups, positions) used the Mann–Whitney *U* test for two groups. Categorical variables were compared using Fisher's exact test where appropriate. All tests were two‐sided with significance threshold set at *p* < 0.05.

## RESULTS

### Cohort characteristics

A total of 242 ACL injuries in 206 unique players were initially identified and met basic inclusion criteria for the RTP analysis (Table 1). Median follow‐up was 2.0 seasons (IQR: 1.0–4.0). For the performance analysis, 105 injuries were excluded: 51 for ACL injuries during their rookie season and 54 for injuries occurring after the 2023 season. This yielded a final performance analysis cohort of 136 ACL injuries in 120 unique players.

**Table 1:** Demographic and injury characteristics of the analysed cohort.

Characteristic	Value
Total ACL injuries	242
Unique players	206
Second ACL injuries	16 (13.3%)
Age at injury, median (IQR)	22.7 (20.4–26.0) years
Pre‐injury experience, median (IQR)	5.0 (3.0–8.0) seasons

*Note*: Second ACL injuries, median age at injury and median pre‐injury experience (seasons played) are included along with the IQRs for median values. Median age at injury was 22.7 (20.4–26.0) years and median pre‐injury experience was 5.0 (3.0–8.0) seasons.

Abbreviations: ACL, anterior cruciate ligament; IQRs, interquartile ranges.

**Figure 1:**
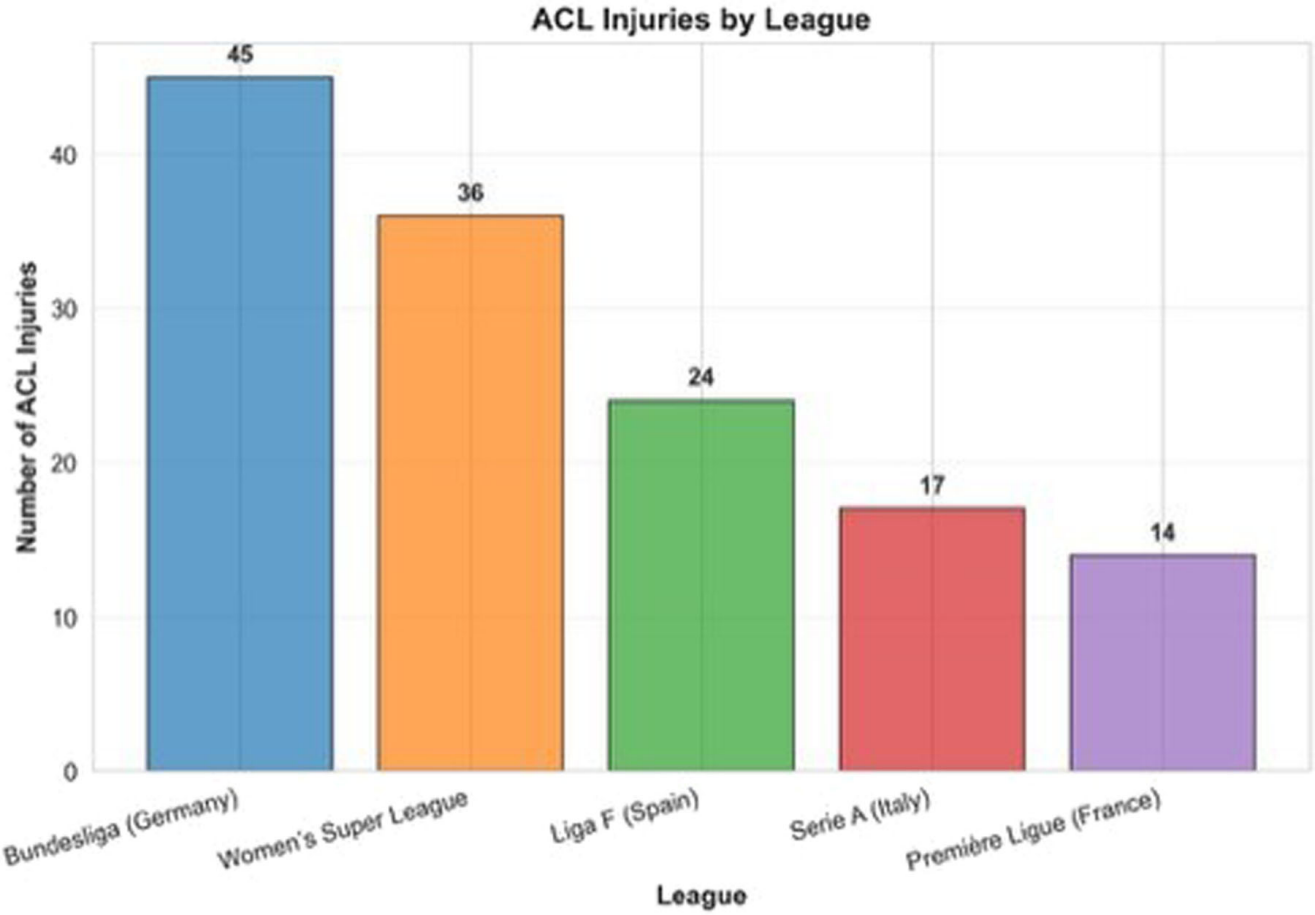
Distribution of ACL injuries across the top five European women's soccer leagues. The German Bundesliga had the highest number of documented cases (33.1%), followed by the English Women's Super League (26.5%), Spanish Primera División Femenina (17.6%), Italian Serie A Women (12.5%) and French Première Ligue (10.3%). ACL, anterior cruciate ligament.

Position distribution showed forwards contributed to the largest proportion of ACL injuries (32.4%), followed by defenders (30.9%), midfielders (28.7%) and goalkeepers (8.1%), broadly consistent with the positional composition of professional rosters. League distribution reflected the relative size and injury reporting quality across leagues (see Figure 1); these proportions should be interpreted in the context of league roster sizes and reporting practices rather than as true incidence rates, as total player counts per league were not uniformly available.

### RTP outcomes

**Figure 2:**
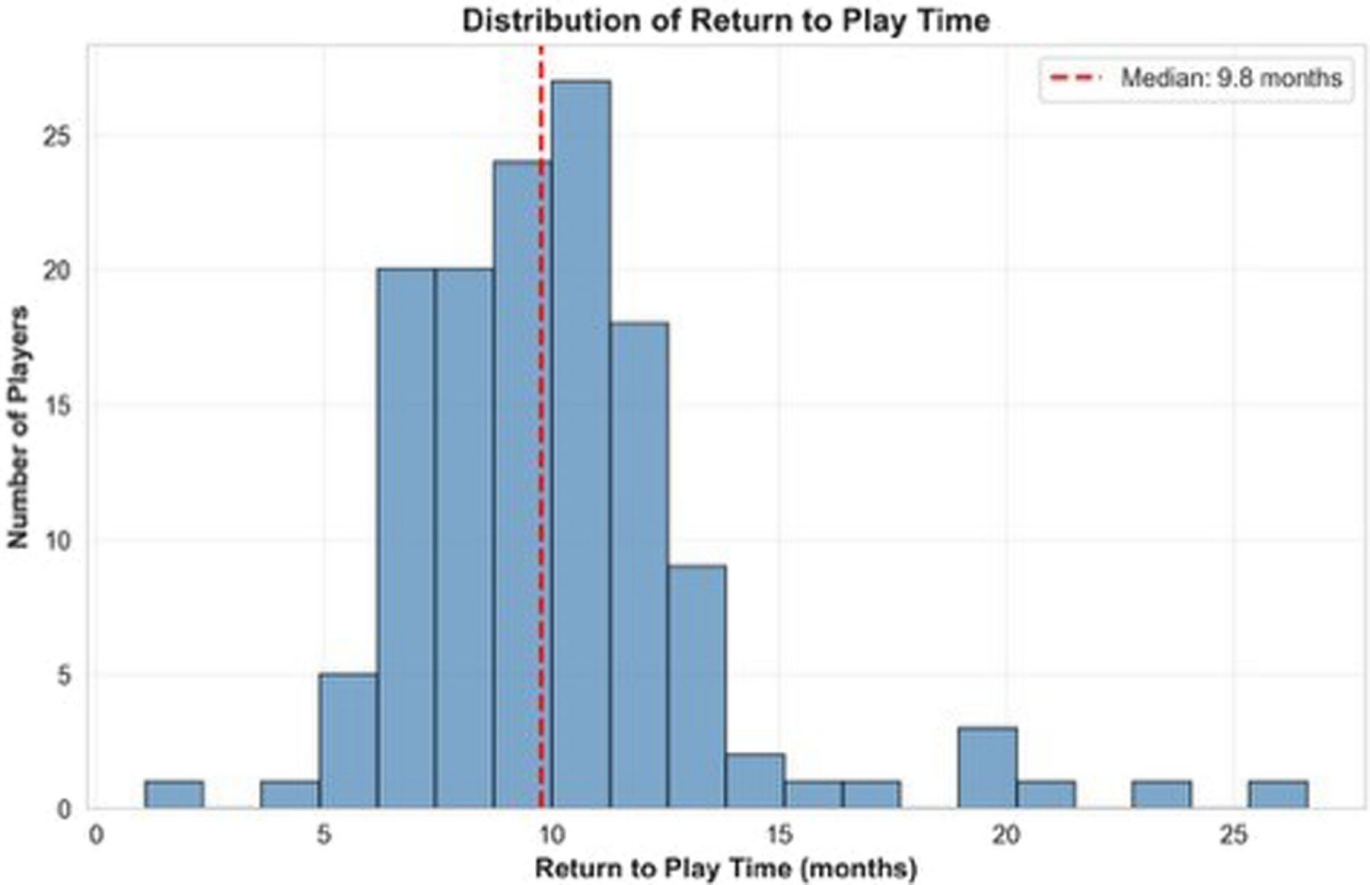
Distribution of return‐to‐play timing following ACLR. The histogram demonstrates that most players returned between 8 and 12 months, with the median return time of 9.8 months (interquartile range: 7.9–11.4). Only 7 players (4.3%) returned before 6 months. ACLR, anterior cruciate ligament reconstruction.

**Figure 3:**
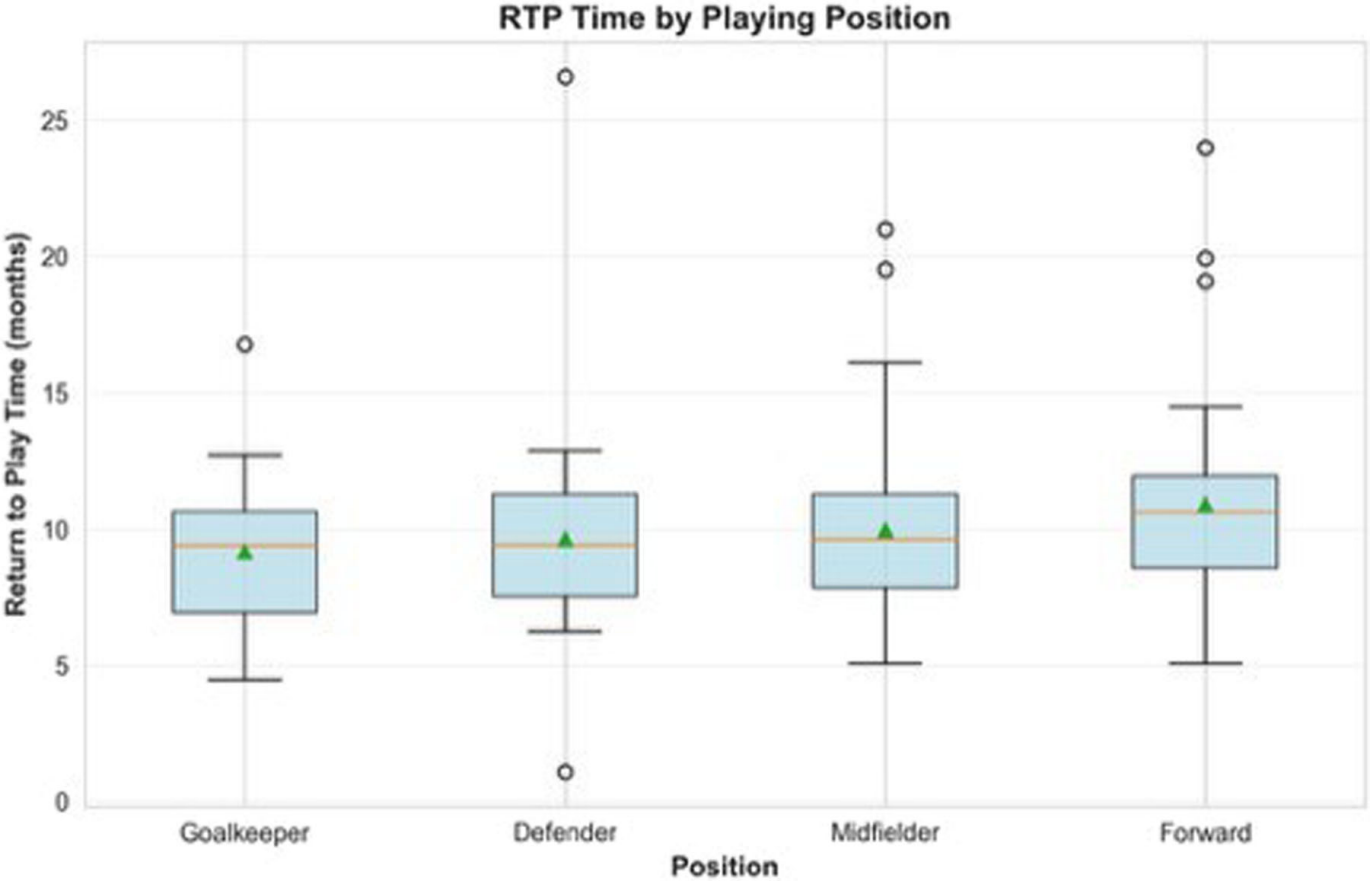
RTP timing by player position. Goalkeepers showed the shortest median return time at 9.4 months (IQR: 6.9–10.6), while forwards had the longest at 10.6 months (IQR: 8.6–11.9). Defenders and midfielders had similar return times of 9.4 and 9.6 months, respectively. IQR, interquartile range; RTP, return‐to‐play.

Of 241 ACL injuries with sufficient data for RTP determination, 161 (66.8%) resulted in RTP, while 80 (33.2%) did not return to competitive play. Median time to RTP was 9.8 months (IQR: 7.9–11.4) (Figure 2). RTP rates varied moderately across leagues (65.8%–70.4%). RTP timing was similar across playing positions, with no significant differences observed (Figure 3). Mean post‐injury career length was ±2.5 seasons (median 2.0, IQR: 2.0–4.0).

### Performance impact analysis

**Figure 4:**
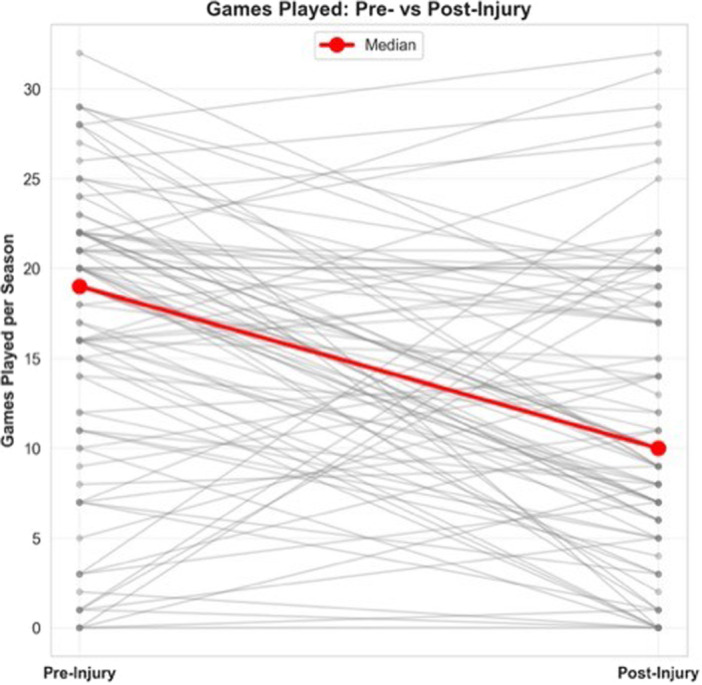
Comparison of games played per season before and after ACLR. Box plots show the distribution of games played in the season before injury versus the first complete season after return to play. There was a significant decrease in median games played from 19.0 to 10.0 games per season (*p* < 0.001). ACLR, anterior cruciate ligament reconstruction.

**Figure 5:**
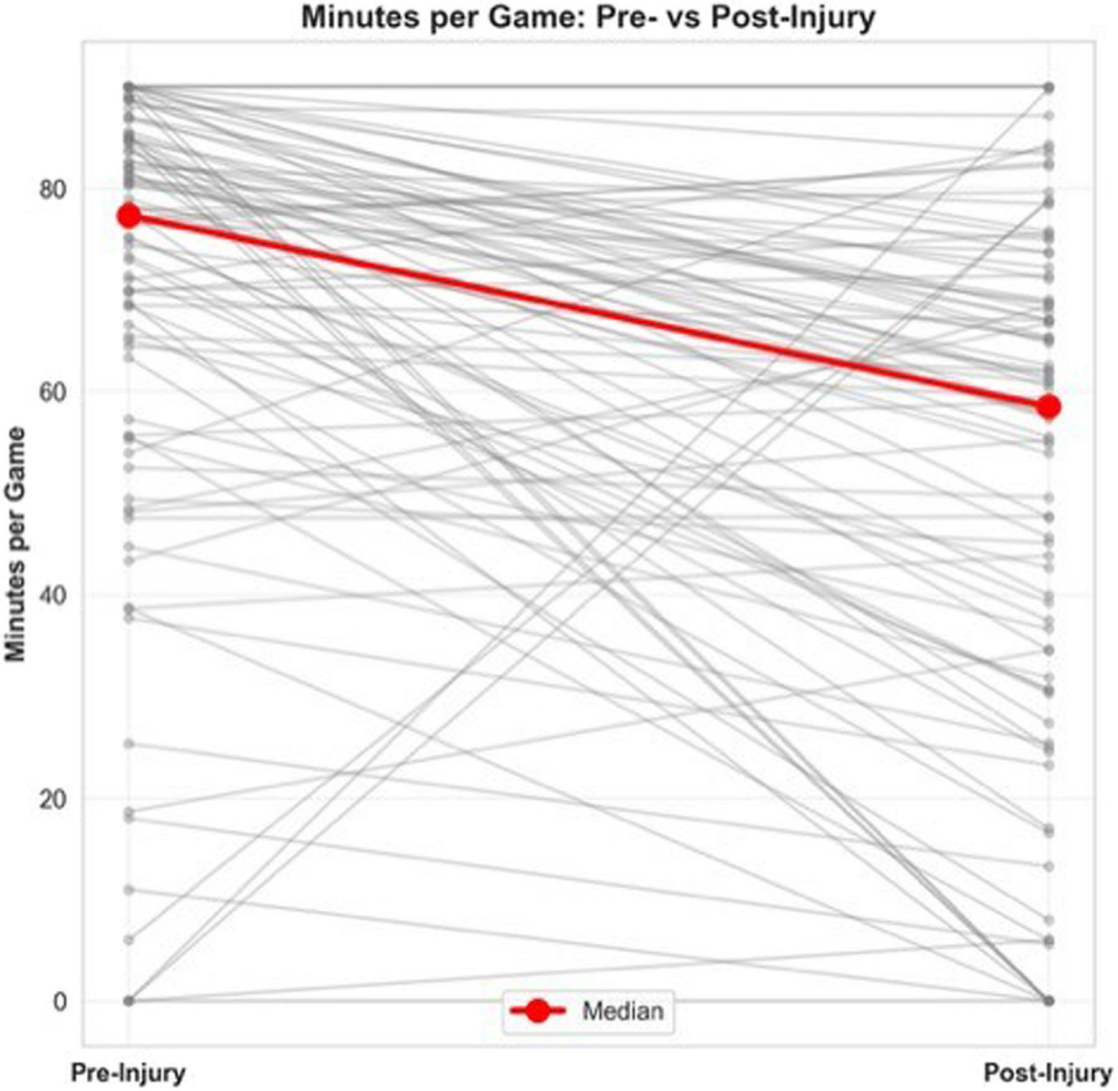
Minutes per game comparison before and after ACLR. The analysis shows a significant decrease in playing time intensity, with median minutes per game declining from 77.3 to 58.5 min (*p* < 0.001), suggesting that players may be managed more conservatively upon return or may face increased competition for playing time. ACLR, anterior cruciate ligament reconstruction.

Following RTP, players demonstrated significant declines in games played per season, total minutes played and minutes played per game, compared with pre‐injury baselines (Figures 4 and 5). Games played, total minutes and minutes per game decreased by median values of 47.4%, 57.8% and 24.4%, respectively (all *p* < 0.001). Significant decreases were observed across all evaluated participation metrics (Table 2).

**Table 2:** Performance comparison: 1 year pre‐ vs. post‐injury (*n* = 90).

Metric	Pre‐injury median (IQR)	Post‐injury median (IQR)	Change (%)	*p* value
Games played	19.0 (11.0–22.0)	10.0 (6.0–18.0)	−47.4%	<0.001
Total minutes	1326 (629–1719)	560 (245–1087)	−57.8%	<0.001
Minutes per game	77.3 (56.0–85.0)	58.5 (31.1–70.5)	−24.4%	<0.001

*Note*: Presented are median values and associated IQRs of games played, total minutes and minutes per game before and after ACLR. Games played, total minutes and minutes per game decreased by median values of 47.4%, 57.8% and 24.4%, respectively (all *p* < 0.001).

Abbreviations: ACLR, anterior cruciate ligament reconstruction; IQRs, interquartile ranges.

### Position‐specific impact

**Figure 6:**
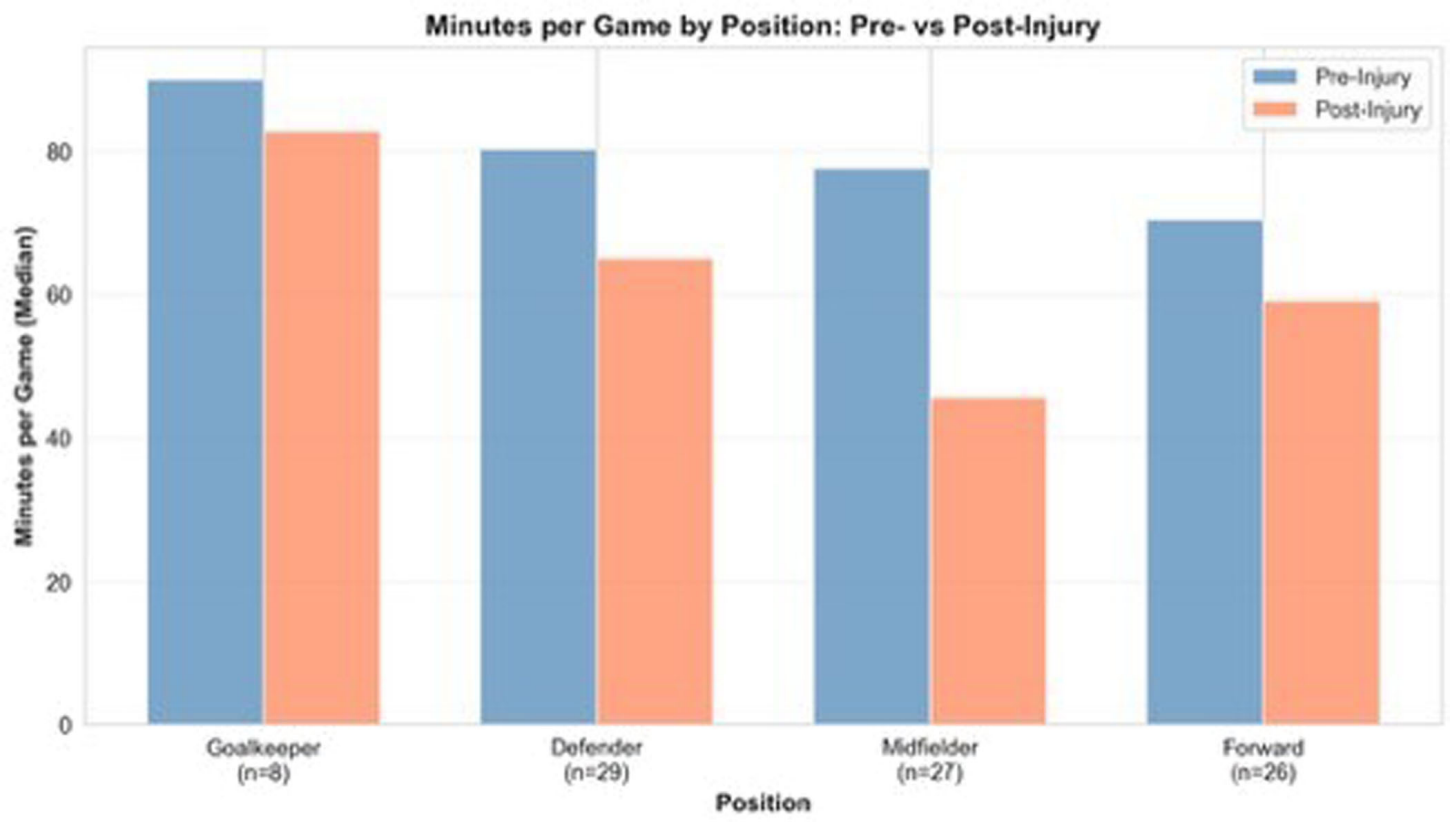
Position‐specific analysis of minutes per game changes following ACLR. Midfielders showed the greatest decline (−41%, *p* < 0.001), followed by defenders (−19%, *p* < 0.01) and forwards (−16%, *p* < 0.01). Goalkeepers experienced minimal change (−8%, *p* = 0.625), likely reflecting the different physical demands of their position. ACLR, anterior cruciate ligament reconstruction.

Analysis of position‐specific performance changes revealed significant variations in the impact of ACL injury and reconstruction. Goalkeepers showed minimal change: 90.0 min/game (IQR: 67.5–90.0) pre‐injury to 82.8 (IQR: 49.0–90.0) post‐injury (*p* = 0.625). Defenders declined from 80.3 (IQR: 55.3–85.3) to 65.1 (IQR: 31.9–74.9), a decrease of 18.9% (*p* = 0.006). Midfielders showed the largest decline, from 77.7 (IQR: 64.2–84.7) to 45.7 (IQR: 24.8–64.8), a decrease of 41.2% (*p* < 0.001). Forwards declined from 70.5 (IQR: 56.0–78.8) to 59.1 (IQR: 35.1–64.5), a decrease of 16.1% (*p* = 0.001). Games played followed similar patterns by position. Goalkeepers: 5.0 (IQR: 1.5–20.5) to 5.5 (IQR: 2.2–10.8) games (*p* = 0.688). Defenders: 16.0 (IQR: 11.0–19.0) to 8.0 (IQR: 5.0–18.0) games (*p* = 0.037). Midfielders: 21.0 (IQR: 15.5–24.5) to 9.0 (IQR: 5.0–18.5) games (*p* = 0.003). Forwards: 19.5 (IQR: 12.5–22.0) to 14.0 (IQR: 9.2–18.0) games (*p* = 0.046) (Figure 6 and Table 3).

**Table 3:** Position‐specific performance change measured by minutes per game.

Position	*n*	Pre‐injury	Post‐injury	Change (%)	*p* value
Goalkeeper	8	90.0 (67.5–90.0)	82.8 (49.0–90.0)	−8.0%	0.625
Defender	29	80.3 (55.3–85.3)	65.1 (31.9–74.9)	−18.9%	0.006
Midfielder	27	77.7 (64.2–84.7)	45.7 (24.8–64.8)	−41.2%	<0.001
Forward	26	70.5 (56.0–78.8)	59.1 (35.1–64.5)	−16.1%	0.001

*Note*: Presented are position‐specific median minutes played per game and associated IQRs before and after ACLR. Percent change in minutes per game and *p* values are given. Midfielders experienced a 41.2% (*p* < 0.001) decrease in minutes per game, while forwards, defenders and goalkeepers showed 16.1% (*p* = 0.625), 18.9% (*p* < 0.01) and 8.0% (*p* < 0.01) decreases, respectively.

Abbreviations: ACLR, anterior cruciate ligament reconstruction; IQRs, interquartile ranges.

### Age‐related analysis

**Figure 7:**
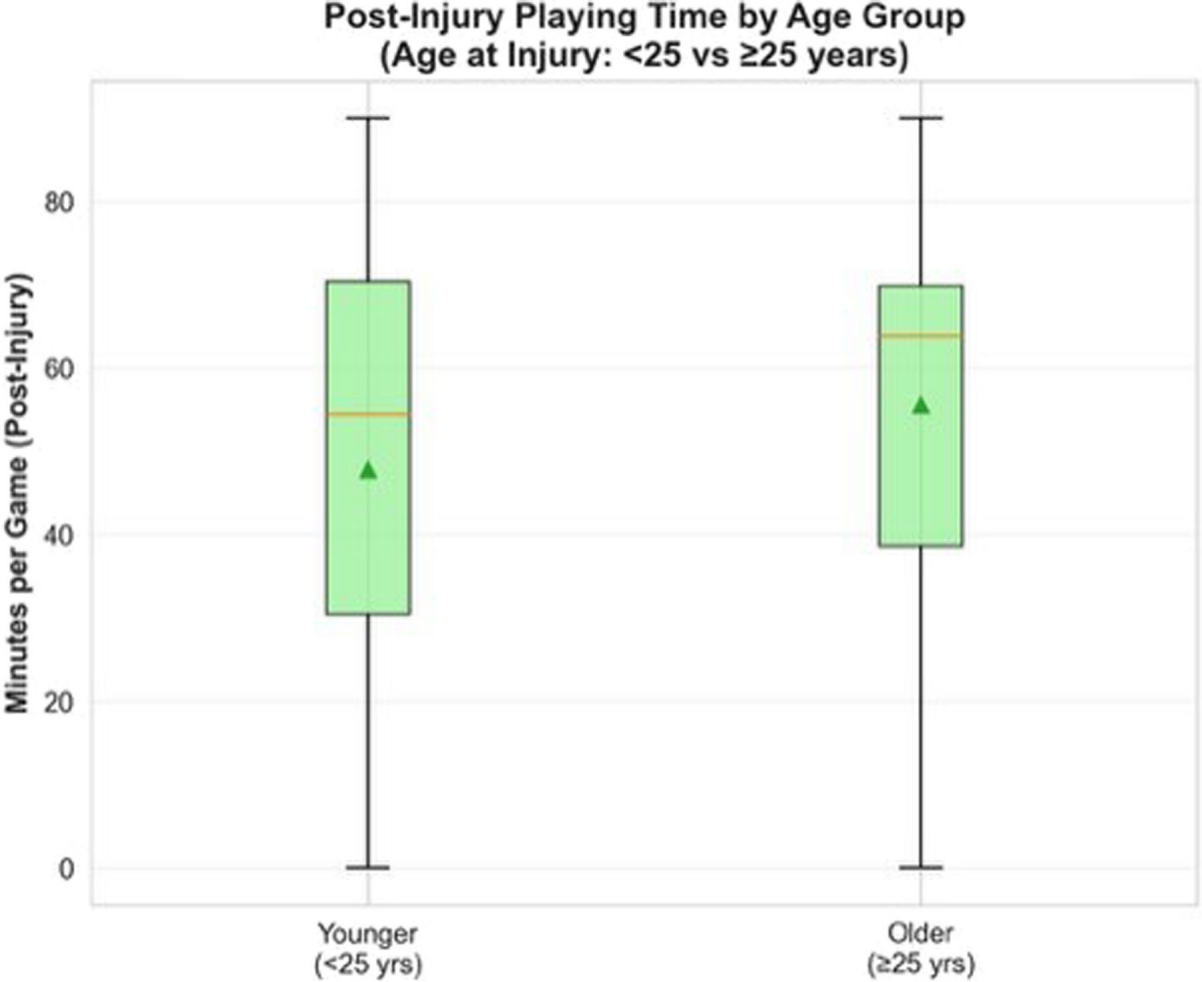
Age‐based comparison of performance outcomes following ACLR. Players were stratified into younger (<25 years) and older (≥25 years) groups. No significant differences were observed between age groups for any performance metric, suggesting that age at injury does not substantially influence short‐term recovery outcomes in elite players. ACLR, anterior cruciate ligament reconstruction.

The younger group (<25 years) had post‐injury minutes per game of 54.3 (IQR: 30.4–70.4), while the older group (≥25 years) had 63.8 (IQR: 38.6–69.8) (Mann–Whitney *U* test, *p* = 0.164). Post‐injury games played were 9.5 (IQR: 5.2–17.8) for younger players and 10.0 (IQR: 6.8–19.2) for older players (*p* = 0.460) (Figure 7).

### Early versus standard RTP

Seven players (5.1%) achieved RTP in less than 6 months (early RTP group), while 129 (94.9%) returned at 6 months or later (standard RTP group). Among early RTP players, position distribution was as follows: one forward, one defender, three midfielders and two goalkeepers. For context, forwards had the longest median RTP time overall (10.6 months) compared to goalkeepers (9.4 months); the small early RTP sample (*n* = 7) precludes position‐specific conclusions within this subgroup. Career longevity was median 3.0 seasons (IQR: 1.0–4.0) for early RTP versus 2.0 seasons (IQR: 2.0–4.0) for standard RTP (Mann–Whitney *U* test, *p* = 0.892). Among early RTP players, 2 of 7 (28.6%) demonstrated 2‐year persistence in elite competition, compared to 49 of 129 (38.0%) for standard RTP (Fisher's exact test, *p* = 0.693). No statistically significant differences in career outcomes were observed between early and standard RTP groups.

## DISCUSSION

This retrospective cohort study examined ACL injuries across five elite European women's soccer leagues, representing one of the largest multi‐league analyses to date, with direct implications for rehabilitation planning, RTP criteria application and performance management in elite players. A RTP rate of 66.8% was observed alongside significant performance decreases in the first season following return, with notable position‐specific variations. The median RTP time of 9.8 months is consistent with current best practice recommendations and emerging evidence supporting more conservative return timelines, while contrasting with reports of some professional male soccer cohorts where earlier returns are more common [13, 15, 17].

Significant reductions in games played, total minutes and minutes per game were observed following ACLR, highlighting the challenges faced by athletes when returning to competition [29]. These findings align with recent studies demonstrating that RTP does not necessarily equate to return to performance and likely reflect a combination of ongoing recovery and subtle performance deficits, competition for playing time and coaching confidence in reintegration [5, 6, 31, 32].

Position‐specific analysis revealed striking differences in the impact of ACL injury across playing roles. Midfielders experienced the most severe decline in performance metrics, possibly reflecting the unique physical and tactical demands of their role and potential reluctance of coaches to reintegrate a player returning from injury. In contrast, goalkeepers demonstrated minimal performance impact, likely due to different movement demands and injury mechanisms [25, 27]. The specialized nature of goalkeeping may also provide more predictable rehabilitation pathways and return criteria. Defenders and forwards showed intermediate declines, suggesting that positional demands influence both recovery and reintegration following ACLR.

The absence of age‐related differences is consistent with recent research in professional athletes, where the elite training environment and medical support may minimize age‐related disparities [23]. Additionally, the relatively narrow age range within elite women's professional soccer (most players between 20 and 30 years) limits the ability to detect age effects [35]. Players remained active for a median of 2.0 seasons following RTP, although this finding should be interpreted cautiously given the potential influence of transfers outside of the leagues studied, retirements and natural career progression [34]. The 91.9% rate of players remaining active at study conclusion suggests that most athletes successfully maintain their professional careers following ACLR [16].

The psychological aspects of ACL recovery, though not directly measured here, likely play a crucial role in the performance patterns observed, particularly through fear of reinjury and kinesiophobia leading to more conservative playing time management [2, 28].

These findings have important implications for rehabilitation and RTP decision‐making in elite women's soccer. Position‐specific rehabilitation strategies, particularly for midfielders, may help address differing position‐specific demands [8]. Medical staff, coaches and athletes should maintain realistic expectations regarding performance recovery and consider gradual reintegration strategies focusing on sport‐specific movements and competitive intensity that can address and potentially limit impacts on performance that may persist beyond RTP [12]. The persistent performance deficits observed also reinforce the importance of ongoing ACL injury prevention efforts in women's soccer and may warrant development of position‐specific prevention protocols given varying injury patterns and recovery challenges observed across playing roles [7, 22].

Future research should include prospective studies with extended follow‐up incorporating surgical technique, rehabilitation protocol details, psychological assessments and biomechanical outcomes. Incorporation of advanced performance metrics, such as Global Positioning System (GPS)‐based movement analysis, may provide more sensitive recovery measures [20]. Development of position‐specific RTP protocols, incorporating sport‐specific movement patterns and role‐relevant testing, represents an important research priority.

### Limitations

Several limitations should be acknowledged when interpreting these findings. The retrospective design limits the ability to control for confounding variables and may introduce selection bias, including survivorship bias, given that performance analyses were restricted to players with sufficient follow‐up data. Performance data availability varied across players and leagues, potentially influencing comparative analyses. The absence of detailed surgical, rehabilitation and psychological data limits the ability to assess technique‐specific or protocol‐specific outcomes. Additionally, the database does not capture players who transferred to leagues outside the five studied following injury, those who retired due to non‐soccer reasons or those who sought treatment in their home country before resuming their career elsewhere, all of which may influence career longevity estimates. Finally, psychological factors, detailed biomechanical assessments and position‐specific player counts per league were not available, representing important areas for future investigation. Performance data availability varied across players and leagues due to differences in reporting standards and career continuity across the five leagues studied.

## CONCLUSIONS

This comprehensive analysis of ACL injuries across elite European women's soccer leagues demonstrates that while many players successfully RTP following ACLR, significant performance impacts persist well into the first year following return. The position‐specific variations observed, particularly the severe impact on midfielders compared to minimal effects in goalkeepers, have important implications for rehabilitation protocols and career management strategies. While RTP rates are encouraging in elite settings, the substantial performance decreases highlight the need for realistic expectations, extended support systems and position‐tailored rehabilitation approaches. These findings emphasize that successful RTP extends beyond simply returning to competition and requires comprehensive strategies to optimize performance recovery and career sustainability following ACLR.

## AUTHOR CONTRIBUTIONS

**Haneef Khan**: Project conception; data collection; data analysis. **Forest Rodriguez:** Data analysis, document writing. **Amina Khan**: Data collection. **Amina Khan** and **Ashley Rosenberg**: Document writing; figure generation. **Stephen Marcaccio**: Project conception; document writing.

## CONFLICT OF INTEREST STATEMENT

The authors declare no conflicts of interest.

## ETHICS STATEMENT

The authors have nothing to report.

## Data Availability

All data are publicly available at soccerdonna.de and in public press reports.
